# Polymorphism, genetic exchange and intragenic recombination of the aureolysin gene among *Staphylococcus aureus *strains

**DOI:** 10.1186/1471-2180-8-129

**Published:** 2008-07-29

**Authors:** Artur J Sabat, Benedykt Wladyka, Klaudia Kosowska-Shick, Hajo Grundmann, Jan Maarten van Dijl, Julia Kowal, Peter C Appelbaum, Adam Dubin, Waleria Hryniewicz

**Affiliations:** 1 National Medicines Institute, 00-725 Warsaw, Poland; 2 Faculty of Biochemistry, Biophysics and Biotechnology, Jagiellonian University, 30-387 Cracow, Poland; 3 Department of Pathology, Hershey Medical Center, P.O. Box 850, Hershey, PA 17033, USA; 4 RijksInstituut voor Volksgezondheid en Milieu (RIVM), Bilthoven, The Netherlands; 5 Department of Medical Microbiology, University Medical Center Groningen and University of Groningen, Groningen, The Netherlands

## Abstract

**Background:**

*Staphylococcus aureus *expresses several proteases, which are thought to contribute to the virulence of this bacterium. Here we focus on aureolysin, the major thermolysin-like metalloprotease. Despite the importance of aureolysin in the physiology and pathogenesis of *S. aureus*, relatively little information was so far available concerning the *aur *gene diversity and mobility within and between the major subdivisions of the *S. aureus *population. Therefore, an epidemiologically and genetically diverse collection of *S. aureus *strains was used to determine the range of aureolysin (*aur*) gene polymorphism.

**Results:**

Sequence analyses support the conclusion that the *aur *gene occurs in two distinct types of related sequences. The *aur *gene was much more polymorphic but, at the same time, showed higher purifying selection than genes utilized for multilocus sequence typing (MLST). Gene trees constructed from *aur *and concatenated MLST genes revealed several putative assortative recombination events (*i.e*. entire *aur *gene exchanges) between divergent lineages of *S. aureus*. Evidence for intragenic recombination events (*i.e*. exchanges of internal *aur *segments) across *aur *genes was also found. The biochemical properties and substrate specificity of the two types of aureolysin purified to homogeneity were studied, revealing minor differences in their affinity to low molecular weight synthetic substrates.

**Conclusion:**

Although numerous nucleotide differences were identified between the *aur *alleles studied, our findings showed that a strong purifying selection is acting on the *aur *gene. Moreover, our study distinguishes between homologous exchanges of the entire *aur *gene (assortative recombination) between divergent *S. aureus *lineages and recombination events within *aur *genes.

## Background

Proteases are important virulence factors for a variety of microbial pathogens and may contribute to tissue degradation and resistance to the host defense system. *Staphylococcus aureus *secretes a typical metalloprotease that is commonly referred to as aureolysin. This enzyme, which is a member of the thermolysin family of zinc-dependent metalloproteases, comprises a single chain of 301 amino acids that consists of a β-pleated N-terminal domain and an α-helical C-terminal domain [1]. *In vitro*, aureolysin activates prothrombin showing pseudocoagulase activity [2] and inactivates mammalian plasma protease inhibitors by cleavage [3,4]. Moreover, the enzyme has been suggested to contribute to the resistance of *S. aureus *to the innate immune system by degradation of the human antimicrobial peptide LL-37 [5] and inhibitory activity against immunoglobulin production by lymphocytes [6]. In addition, aureolysin is required for activation of a serine protease (SspA; also named V8 protease) secreted by the same microorganism [7-9]. Recently, Beaufort and colleagues [10] reported that aureolysin is capable of converting the zymogen pro-urokinase into its active form. Urokinase, in turn, is a component of the plasminogen activation system. Upon activation, plasmin displays enzymatic activity towards a number of substrates, including fibrin. Therefore, aureolysin may contribute to activation of the host's fibrinolytic system by *S. aureus*, and thus promote bacterial spread and invasion. Nevertheless, in a mouse model of septic arthritis, inactivation of *aur *gene did not affect the frequency or the severity of joint disease [11].

In our previous study, we cloned the aureolysin gene and proposed the name *aur *[12]. Sequence analysis revealed a coding sequence (CDS) of 1,527 nucleotides, starting with a GTG codon with the potential of encoding the aureolysin preproenzyme of 509 amino acids. The nascent translation product of the *aur *gene included an N-terminal signal peptide (prefragment) of 27 amino acids with a typical signal peptidase cleavage site, followed by a profragment and a mature protease composed of 181 and 301 residues, respectively. We showed the presence of the *aur *gene in all investigated community-acquired as well as laboratory strains [12]. PCR-restriction fragment length polymorphism (PCR-RFLP) analysis of the *aur *gene revealed only two RFLP types [12]. Sequencing of the *aur *gene from two representative strains of each of those two PCR-RFLP types confirmed that the gene occurred in two forms (type I and type II). Deduced amino acid sequences of aureolysin were almost identical within a type, whereas the sequence identity between the two types of genes was only 89%. The strong conservation of *aur *among two types of related sequences argues in favor of the possibility that aureolysin has important housekeeping functions.

Introduction of the multilocus sequence typing (MLST) method [13], which measures sequence variation at seven slowly evolving housekeeping genes, has resulted in a detailed understanding of *S. aureus *population structure. The evolution of *S. aureus *may have been influenced by chromosomal replacements [14]. Nevertheless, the MLST data have revealed a predominantly clonal evolution of this species and its subdivision into two fundamental groups (supergroups) consisting of multiple clonal complexes [15]. Although MLST has been primarily developed as a typing approach, it has also been used successfully for examining the degree to which homologous recombination has occurred within the *S. aureus *genome [14,16].

Our previous study, which focused on aureolysin [12], was conducted shortly before the MLST era. At the time, it was felt that the presence of only two types of *aur *genes could reflect the existence of two supergroups of *S. aureus *strains. However, a real degree of polymorphism of the *aur *gene could not be determined previously, since we used only local strains and therefore a very limited number of genotypes. In the current study, we have therefore focused attention on the genetic analysis of the *aur *gene to determine its diversity and the extent to which it was mobile within and between the major population subdivisions. For this purpose, we have used epidemiologically and genetically diverse strains of *S. aureus*. Additionally, we performed biochemical studies on aureolysins of type I and type II to determine their properties such as enzymatic specificity and activity.

## Methods

### Bacterial strains

For *aur *sequencing, a group of 24 methicillin-resistant *S. aureus *(MRSA) and 42 methicillin-susceptible *S*. *aureus *(MSSA) epidemiologically unrelated strains was selected from different collections of *S. aureus *strains [22,23]. Strain selection was based on preliminary MLST data and was intended to include strains with a large degree of diversity. Strains were derived from a variety of human infections as well as from nasal swabs of asymptomatic carriers. Their geographic origins included mainly European countries but also the first three vancomycin-resistant *S. aureus *(VRSA) strains, MI-VRSA (strain VRS1, HIP11714), PA-VRSA (strain VRS2, HIP11983) and NY-VRSA (strain VRS3, HIP13170), recovered in the United States were included in this study. Informed consent was obtained from all patients and participants which were involved in our study. All procedures were performed according to guidelines of the local ethics committees which are in compliance with the Helsinki Declaration. All strains were coded to protect patients and participants confidentiality. Additionally, thirteen *S. aureus *strains for which the genome sequences are available on the NCBI website (see Availability and requirements section for URL) were analyzed.

For studying biochemical properties of the two types of aureolysin, strains V8-BC10 (type I) and the *sarA *and *sspA *double mutant of 8325-4 (type II) (obtained from Dr S.O. Arvidson, Karolinska Institute, Stockholm [8], and Dr S. Foster, University of Sheffield, Sheffield, UK, respectively [11]) were used.

### MLST

MLST was performed according to protocol developed by Enright *et al*. [13]. Sequences of each locus were compared to the data in the *S*. *aureus *MLST database (see Availability and requirements section for URL), and resulting allelic profiles were assigned to particular sequence types (STs) for each strain. Sequences of unknown alleles and the allelic profiles of novel STs were submitted to the MLST database curator for allele or ST number assignment. BURST software (see Availability and requirements section for URL) was used to classify related STs into clonal complexes (CCs) of phylogenetic relationships. Such clusters were composed of two or more STs which differed at a single locus (single-locus variants) or two loci (double-locus variants) [15]. A singleton was defined as a sequence type that is not grouped into a clonal complex.

### Sequence analysis of *aur *genes

PCR products were used as the templates for sequencing reactions. These templates covering the whole *aur *gene were generated by use of recombinant *Taq *DNA polymerase (Fermentas, Vilnius, Lithuania) (Cycling conditions: denaturation, 94°C/30 s; annealing, 55°C/30 s; elongation, 72°C/30 s) with primer pairs *aur*-F1 (5'-GCTGTTTTTAARATTTCAGGAGG) and *aur*-R1 (5'-TGAAATTTCTGGTGTYACTGTAATTAA), *aur*-F2 (5'-GAAATCGATGGTGACAGTAATAA) and *aur*-R2 (5'-CTACGTCATTTGCACCYGATAA), and *aur*-F3 (5'-GACAAAATGATYTATGGTGATGG) and *aur*-R3 (5'-GTGTTTAACATTACTTCTTCTTGTTT). The same primers were used for the sequence reactions.

### Analysis of nucleotide and amino acid sequence data

Nucleotide sequences were aligned using ClustalW (see Availability and requirements section for URL) software. Nucleotide diversity was calculated using the DnaSP package, version 4.10 [24]. The proportion of synonymous (silent; *ds*) and non-synonymous (amino acid-changing; *dn*) substitution rates was calculated using the software SNAP (see Availability and requirements section for URL). If purifying selection has occurred, a gene has a *ds*/*dn *ratio > 1. Absence of selection should generate *ds *= *dn*. A value < 1 indicates diversifying selection or accelerated evolution. Neighbor-joining (NJ) trees were constructed using the program Mega version 3.1 [25]. Genetic distances were calculated by the Kimura two-parameter model. Bootstrap analysis (1000 repeats) was performed to evaluate the topology of the phylogenetic tree. Recombinant *aur *sequences were detected employing the RDP-V2 Beta 08 software [26] using six automated recombination detection methods including RDP [27], Geneconv [28], Bootscanning [29], Maximum Chi Square (MaxChi) [30], Chimaera [31], and Sister Scanning (SiScan) [32]. Results were then checked by visual inspection. Amino acid sequences were edited in Genedoc software (version 2.6.02) [33].

### Purification of aureolysins

*S. aureus *V8-BC10 and the *sarA *and *sspA *double mutant of 8325-4 were grown in Tryptic Soy Broth (Fluka Chemie AG, Buchs, Switzerland), supplemented with erythromycin 10 mg l^-1 ^and neomycin 50 mg l^-1 ^in case of the latter strain, for 15 h at 37°C. Proteins from clarified culture fluids were precipitated with ammonium sulfate (80% saturation), recovered by centrifugation (15 000 g, 1 h, 4°C), dialyzed against 20 mM Tris HCl pH 7.8 supplemented with 10 mM CaCl_2 _(buffer A), and loaded onto Q-Sepharose FF column equilibrated with buffer A. Proteins were eluted from the column using increasing NaCl linear gradient in buffer A, and fractions exhibiting EDTA-sensitive proteolytic activity were pooled. The above procedure resulted in an electrophoreticaly homogenous protein. N-terminal protein sequence analysis was performed at BioCentrum Ltd. (Krakow, Poland)

### Comparison of biochemical properties of aureolysins

Specific activity of enzymes was determined by α-2-macroglobulin (α-2 M) (BioCentrum Ltd.) titration. Constant amounts of enzymes (0.02 nmol) were incubated with increasing amounts of α-2 M for 1 h, at room temperature. Then, high molecular weight protein substrate (Hide Power Azure, Calbiochem) was added and residual enzyme activity was measured as an increase of absorbance at 595 nm after 30 min incubation at 37°C. It was assumed that one molecule of α-2 M inhibits two molecules of aureolysin. For kinetic studies low molecular weight synthetic substrates, Fa-Gly-Ala-NH_2_, Fa-Gly-Val-NH_2_, Fa-Gly-Phe-NH_2 _and Fa-Gly-Leu-NH_2_, (Bachem, Switzerland), were used. The kinetic constants were determined from the initial rates of hydrolysis by the Lineweaver-Burk method, based on triplicate rate determinations at five substrate concentrations in the range of 0.1–5 mM. Galanin digestion was performed by incubation of the substrate with the enzymes in 2000:1 molar ratio for 5 min, 37°C, and nano-LC-MS/MS analysis of the obtained products were done at Faculty of Chemistry and Regional Laboratory, Jagiellonian University, as describe elsewhere [34]. To compare degradation patterns of proteins by aureolysins, α-1-antitrypsin, α-1-antichymotrypsin (both from BioCentrum), collagen, beta casein (both from Sigma), HMWK and LMWK, were incubated with the same amounts of active aureolysins of type I and type II at the molar ratio from 5:1 to 200:1 for the time ranging from 5 min to 12 hours. Then, EDTA was added and reaction mixtures were subjected to high throughput electrophoresis [35].

### Nucleotide sequence accession numbers

Nucleotide sequences for the *aur *gene can be found in the GenBank database under accession numbers EF070219 to EF070241.

## Results

### Clustering of *S. aureus *strains by MLST

**Figure 1:**
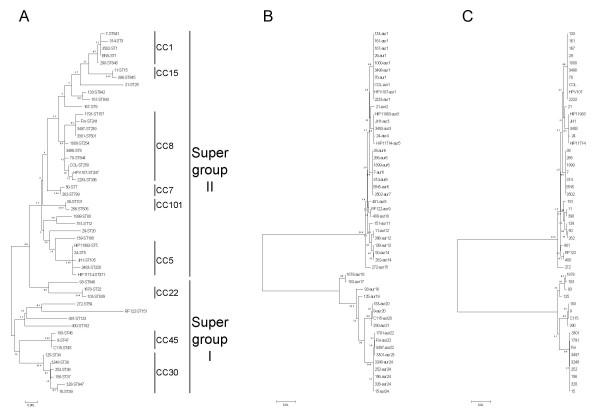
**Phylogenetic trees based on (A) MLST, (B) entire *aur *genes and (C) shortened *aur *sequences**. Phylogenetic trees for 50 *S. aureus *strains were constructed by using the neighbor-joining method based on the Kimura two-parameter model with MEGA 3.1. All branch lengths are drawn to scale. Bootstrap values were calculated for 1,000 replicates and are given for each branch. Strains used represent all combinations of STs and *aur *alleles. Each strain is abbreviated with the isolate designation, followed by the MLST (ST) designation in panel A, and the *aur *allele designation in panel B. CCs and supergroups are indicated in panel A. Phylogenetic analysis based on housekeeping gene sequences placed strains in groups which corresponded to CCs (Table 1) identified by the BURST analysis with the exception of the ST188 strains which were sorted as unique (panel A). The Supergroup is based on a reconstruction of *S. aureus *phylogeny conducted in this study and those presented previously [17,18].

A total of 79 strains revealed 48 different sequence types (STs) on the basis of the MLST data (Table 1). On the basis of BURST analysis, thirty six STs with 65 strains were grouped into nine CCs, while the remaining twelve STs that comprised 14 strains were singletons. Sequences of seven MLST housekeeping genes were concatenated to further examine the relationship between the sequence type and the *aur *allele. The resulting gene tree (Figure 1A) was consistent with the one reported previously and supports the division of *S. aureus *strains into two fundamental groups (supergroups I and II) which comprise multiple clonal complexes [15]. Supergroups reflect the evolutionary relationships among the strains of *S. aureus *and are concordant to subspecies groups distinguished by Robinson and colleagues [17] and two main groups developed by Cooper and Feil [18].

**Table 1:** Characteristics of 79 *S. aureus *strains used in this study, grouped according to the BURST analysis.

**Strain**	**Origin**	**CC^a^**	**ST**	***arcc***	***aroe***	***glpf***	***gmk***	***pta***	***tpi***	***yqil***	***aur***	**Methicillin^b^**
133	England	97	842	3	1	1	1	1	5	38	1	MSSA
161	England	NA	843	3	1	31	1	29	5	3	1	MSSA
167	England	NA	9	3	3	1	1	1	1	10	1	MSSA
29	England	20	20	4	9	1	8	1	10	8	1	MSSA
1000/93	Germany	8	254	3	32	1	1	4	4	3	1	MRSA
3498	Russia	8	8	3	3	1	1	4	4	3	1	MRSA
USA300	USA	8	8	3	3	1	1	4	4	3	1	MRSA
3521	Lithuania	8	8	3	3	1	1	4	4	3	1	MRSA
399	England	8	8	3	3	1	1	4	4	3	1	MSSA
364	England	8	8	3	3	1	1	4	4	3	1	MSSA
NCTC 8325	England	8	8	3	3	1	1	4	4	3	1	MSSA
Newman	England	8	8	3	3	1	1	4	4	3	1	MSSA
70	England	8	844	3	51	1	1	4	4	3	1	MSSA
COL	England	8	250	3	3	1	1	4	4	16	1	MRSA
HPV107	Portugal	8	247	3	3	1	12	4	4	16	1	MRSA
2260	Poland	8	247	3	3	1	12	4	4	16	1	MRSA
2233	Poland	8	336	3	73	1	12	4	4	16	1	MRSA
1791	Poland	8	157	2	3	26	1	4	39	3	22	MRSA
Fin	Finland	8	241	2	3	1	1	4	4	30	22	MRSA
3497	Bulgaria	8	239	2	3	1	1	4	4	3	22	MRSA
H390	Poland	8	239	2	3	1	1	4	4	3	22	MRSA
3254	Poland	8	239	2	3	1	1	4	4	3	22	MRSA
3301	Lithuania	8	501	65	3	1	1	4	4	3	23	MRSA
21	England	25	25	4	1	4	1	5	5	4	2	MSSA
57	England	25	25	4	1	4	1	5	5	4	2	MSSA
116	England	25	25	4	1	4	1	5	5	4	2	MSSA
HIP11983	USA	5	5	1	4	1	4	12	1	10	3	VRSA
HIP13170	USA	5	5	1	4	1	4	12	1	10	3	VRSA
N315	Japan	5	5	1	4	1	4	12	1	10	3	MRSA
Mu50	Japan	5	5	1	4	1	4	12	1	10	3	MRSA
Mu3	Japan	5	5	1	4	1	4	12	1	10	3	MRSA
JH1	USA	5	105	1	4	1	4	12	1	28	3	MRSA
JH9	USA	5	105	1	4	1	4	12	1	28	3	MRSA
3483	Slovenia	5	228	1	4	1	4	12	24	29	3	MRSA
24	Poland	5	5	1	4	1	4	12	1	10	4	MRSA
HIP11714	USA	5	371	1	4	53	4	12	1	10	5	VRSA
38	England	101	101	3	1	14	15	11	19	3	6	MSSA
266	England	101	505	24	1	14	15	11	19	3	6	MSSA
1899	Poland	80	80	1	3	1	14	11	51	10	6	MRSA
7	England	1	841	1	1	25	1	1	1	1	6	MSSA
314	England	1	3	1	1	1	9	1	1	12	6	MSSA
BN5	Poland	1	1	1	1	1	1	1	1	1	6	MRSA
MW2	USA	1	1	1	1	1	1	1	1	1	6	MRSA
476	England	1	1	1	1	1	1	1	1	1	6	MSSA
3502	Bulgaria	1	1	1	1	1	1	1	1	1	7	MRSA
139	England	1	188	3	1	1	8	1	1	1	13	MSSA
380	England	1	188	3	1	1	8	1	1	1	13	MSSA
290	England	1	848	10	1	1	1	12	1	1	21	MSSA
481	England	121	123	6	5	6	2	7	17	19	8	MSSA
RF122^c^	Ireland	NA	151	6	72	12	43	49	67	59	9	MSSA
400	England	NA	182	18	18	6	2	13	15	18	10	MSSA
151	England	12	12	1	3	1	8	11	5	11	11	MSSA
11	England	15	15	13	13	1	1	12	11	13	12	MSSA
794	Poland	15	15	13	13	1	1	12	11	13	12	MSSA
245	England	15	15	13	13	1	1	12	11	13	12	MSSA
398	England	15	845	13	13	1	1	12	34	13	12	MSSA
50	England	7	7	5	4	1	4	4	6	3	14	MSSA
262	England	7	789	3	4	1	4	4	6	3	14	MSSA
271	England	7	789	3	4	1	4	4	6	3	14	MSSA
272	England	59	59	19	23	15	2	19	20	15	15	MSSA
1678/96	Germany	22	22	7	6	1	5	8	8	6	16	MRSA
136	England	22	22	7	6	1	5	8	8	6	16	MSSA
160	England	22	22	7	6	1	5	8	8	6	16	MSSA
103	England	22	839	7	6	112	5	8	8	6	17	MSSA
93	England	NA	846	7	6	28	27	11	40	43	18	MSSA
183	England	45	46	10	14	8	6	14	3	2	20	MSSA
299	England	45	46	10	14	8	6	14	3	2	20	MSSA
9	England	45	47	10	11	8	6	10	3	2	20	MSSA
87	England	45	47	10	11	8	6	10	3	2	20	MSSA
C115	Poland	45	45	10	14	8	6	10	3	2	20	MRSA
A005a	Poland	45	45	10	14	8	6	10	3	2	20	MSSA
125	England	30	34	8	2	2	2	6	3	2	19	MSSA
3248	Czech	30	30	2	2	2	2	6	3	2	24	MRSA
2956	Poland	30	30	2	2	2	2	6	3	2	24	MRSA
436	England	30	30	2	2	2	2	6	3	2	24	MSSA
252	England	30	36	2	2	2	2	3	3	2	24	MRSA
156	England	30	37	2	2	2	2	15	3	2	24	MSSA
328	England	30	847	2	46	24	2	2	2	2	24	MSSA
15	England	30	39	2	2	2	2	2	2	2	24	MSSA

^a ^NA indicates that the strain may differ by at least three MLST genes from other strains recorded in the MLST database as of January 2008.

^b ^VRSA, vancomycin-resistant MRSA

^c ^RF122 was the only strain of animal origin analyzed in this study and was isolated from bovine mastitis

### Sequence analysis of the *aur *gene

**Figure 2:**
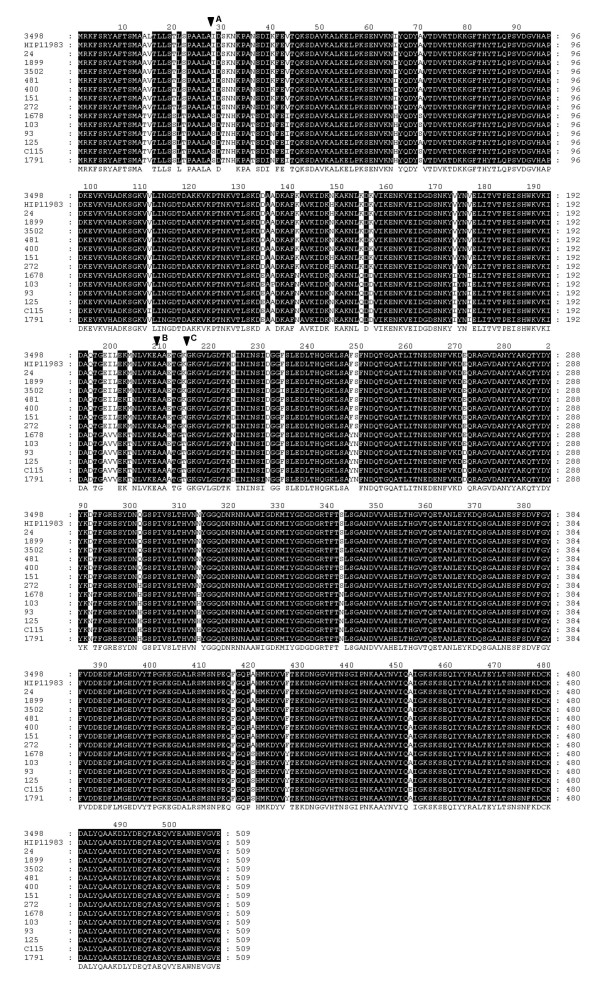
**Sequence comparison of metalloproteases from *S. aureus***. The deduced amino acid sequence represented by: strain 3498 is shared by alleles *aur1 *and *aur2*; strain HIP11983 is shared by alleles *aur3 *and *aur5*; strain 24 is characteristic for allele *aur4*; strain 1899 is characteristic for allele *aur6*; strain 3502 is characteristic for allele *aur7*; strain 481 is characteristic for allele *aur8*; strain 400 is shared by alleles *aur9*, *aur10 *and *aur12*; strain151 is shared by alleles *aur11*, *aur13 *and *aur14*; strain 272 is characteristic for allele *aur15*; strain 1678 is characteristic for allele *aur16*; strain103 is characteristic for allele *aur17*; strain 93 is shared by alleles *aur18 *and *aur24*; strain 125 is characteristic for allele *aur19*; strain C115 is shared by alleles *aur20 *and *aur21*; strain 1791 is shared by alleles *aur22 *and *aur23*. Black shading indicates identical amino acids. The arrowhead A indicates the predicted site of cleavage by signal peptidase; the arrowhead designated B shows the cleavage site of properly processed mature metalloprotease; and arrowhead C shows the cleavage site of the truncated mature form. The deduced amino acid sequences for strains V8-BC10 [12] and NCTC 8325  are identical to those for strains 93 and 3498, respectively. The figure was generated with Genedoc software (version 2.6.02) [33].

Nucleotide sequences obtained during this study were aligned with those *aur *sequences which are available on the NCBI website ((see Availability and requirements section for URL), 6 January 2008) (strains 8325, Newman, COL, MRSA252, MSSA476, MW2, Mu3, Mu50, N315, USA300, RF122, JH1, and JH9). Sequence analysis of the *aur *gene confirmed that all investigated MRSA and MSSA strains possessed the *aur *gene of the same length of 1530 bp. Deletion or insertion events as well as truncation of the aureolysin protein by introduction of an early termination codon were not found. The phylogenetic relationships of the *aur *sequences are shown in Figure 1B. We found twenty four nucleotide alleles of the *aur *gene (Table 2) which translated to 15 amino acid sequences (Figure 2). Sequencing analysis supports our conclusion [12] that the *aur *gene occurs in two distinct groups (type I and type II) of related sequences. However, we found a greater diversity of alleles than reported previously [12]. This was not unexpected due to a much more diverse strain collection. There were 195 (12.7%) polymorphic nucleotide sites, which resulted in 43 (8.4%) variable inferred amino acid positions. Type I consisted of 9 nucleotide alleles which translated to 6 amino acid sequences, and type II consisted of 15 nucleotide alleles which translated to 9 amino acid sequences. Pairwise differences in nucleotide sequences between representative pairs of alleles ranged from 1 to 40 (0.1–2.6%) nucleotide sites within type I, and from 1 to 13 (0.1–0.8%) nucleotide sites within type II, whereas between the types the differences ranged from 146 to 170 nucleotide sites (9.5–11.1%). The deduced amino acid diversity of preproaureolysin varied from 1 to 6 (0.2–1.2%) amino acid positions within type I, and from 1 to 4 (0.2–0.8%) amino acid positions within type II, whereas between types the difference was from 31 to 38 (6.1–7.5%) amino acid positions.

**Table 2:** Sequence parameters for 7 MLST housekeeping and *aur *genes.

Gene	Sequence length (bp)	No. of alleles	No. of polymorphic sites	Nucleotide diversity	Purifying selection (ds/dn)
*arcC*	456–457	14	15	0.0085	6.53
*aroE*	456	17	32	0.0146	7.91
*glpF*	465	15	18	0.0074	7.39
*gmk*	429	12	12	0.0102	10.50
*pta*	474	17	19	0.0079	6.41
*tpi*	402	19	27	0.0123	6.34
*yqiL*	516	20	32	0.0109	7.44
aur-full length	1530	24	195	0.0554	17.56
aur-mature	906	20	99	0.0459	27.05
aur-profragment	543	17	86	0.0711	14.88
aur-prefragment	81	5	10	0.0556	5.49

### Molecular evolution

Data reporting numbers of alleles for all genes are summarized in Table 2. We found that the *aur *gene was much more polymorphic than the housekeeping genes. The nucleotide diversity value for the *aur *gene was 0.0554 and about five times greater than those for MLST housekeeping genes, ranging from 0.0074 to 0.0146 (mean = 0.0103). This implies that the *aur *gene was evolving at a higher rate than the MLST housekeeping genes (Table 2). However particular fragments of the *aur *gene showed different values of diversity. The most polymorphic was the profragment (0.0711) and the least polymorphic was the fragment encoding the mature protein (0.0459); by comparison, the prefragment showed a value (0.0556), which is almost identical to that of the whole gene.

Most polymorphisms led to synonymous substitutions indicating a role of an environmental purifying selection in the evolution. The strength of purifying selection, expressed as the *ds/dn *ratio, showed a clear difference between the MLST housekeeping genes, between 6.34 and 10.50 (mean *ds/dn *= 7.50) and the *aur *gene, *ds/dn *= 17.56. Thus, there was a higher purifying selection in the *aur *gene; however particular regions of the gene showed different purifying strengths. The highest value was observed for the region encoding the mature enzyme, *ds/dn *= 27.05. The purifying selection for the region encoding the profragment was also higher, *ds/dn *= 14.88, than those for the MLST housekeeping genes and values were between those for the mature protein and the MLST genes. The (pre)fragment coding for the signal peptide had a value, *ds/dn *= 5.49, which was similar to those for the MLST genes.

### Evidence for homologous exchange of the entire *aur *gene (assortative recombination) between the supergroups

Homologous exchange of the *aur *gene between the supergroups leads to the presence of *aur *alleles of the same type in strains from both supergroups. In general, strains of supergroup I and II had *aur *of type I and type II, respectively. However, there were also some exceptions, which indicated the transfer of the *aur *gene. For *aur *of type I (alleles *aur16 *through *aur24*), the allele *aur21 *was found in a strain belonging to ST848 which in turn belonged to complex CC1. The allele *aur22 *was found in strains of ST157, ST241, and ST239 which were a part of CC8; the allele *aur23 *was found in the ST501 strain which was a member of CC8. Strains of clonal complexes CC1 and CC8 were representatives of the MLST supergroup II. For *aur *of type II (alleles *aur1 *through *aur15*), the alleles *aur8*, *aur9*, *aur10*, and *aur15 *were found in strains of ST123, ST151, ST182, and ST59, respectively, which were singletons and representatives of the MLST supergroup I.

### Evidence for homologous exchange of the entire *aur *gene (assortative recombination) within a supergroup

To examine the possibility of homologous exchange within a supergroup, we determined genetic variability of the *aur *gene within a sequence type and a clonal complex. We excluded from the analysis those strains for which we proved the *aur *transfer between the supergroups (strains of ST848, ST157, ST241, ST239, ST501, ST123, ST151, ST182, and ST59). Strains of the same ST usually possessed an identical *aur *allele with the exception of ST1 (*aur6 *and *aur7*) and ST5 (*aur3 *and *aur4*); however, their *aur *alleles differed by only a single nucleotide. With the exception of CC1 and CC101, strains of a given MLST clonal complex possessed their own *aur *alleles. In general, within a clonal complex, *aur *alleles were either identical (CC8, CC101, CC15, CC7, CC45) or showed little variation differing by a single nucleotide (CC1, *aur6 *and *aur7*; CC5, *aur3 *and *aur4*, and *aur3 *and *aur5*; CC22, *aur16 *and *aur17*) or by two nucleotides (CC5, *aur4 *and *aur5*). The *aur *alleles of different CCs differed by 14 to 38 nucleotides in supergroup I, and 4 to 13 nucleotides in supergroup II. Therefore, STs within a clonal complex should have identical *aur *alleles or alleles differing by up to two nucleotides whereas those *aur *alleles in strains of STs which are not related by MLST should differ between each other by at least 4 nucleotides. Using these rules, we detected a single event of homologous exchange within the supergroup I and numerous events of such transfer within the supergroup II. Exceptional cases of variation greater than two nucleotides of difference between the *aur *alleles within a clonal complex were found in CC1 (supergroup II) and CC30 (supergroup I). In these two clonal complexes almost all STs had an identical *aur *allele within a given clonal complex (CC1, *aur6*; and CC30, *aur24*). However, ST188 (CC1) and ST34 (CC30) which possessed the allele *aur13 *and *aur19*, respectively, differed substantially by 6 nucleotides from the allele *aur6 *and 16 nucleotides from the allele *aur24*. In supergroup II, several STs not related by MLST had an identical *aur *allele or closely related *aur *alleles (differing by a single nucleotide). Strains of ST842, ST843, ST9, ST20 and CC8 possessed the allele *aur1*. Strains of CC101, ST89 and almost all CC1 possessed the allele *aur6 *and strain 3502 (ST1) had *aur7*; ST151 and ST182 possessed alleles *aur9 *and *aur10*, respectively and ST188 and CC7 possessed alleles *aur13 *and *aur14*, respectively.

### Evidence for mosaic structure among the *aur *alleles

**Figure 3:**
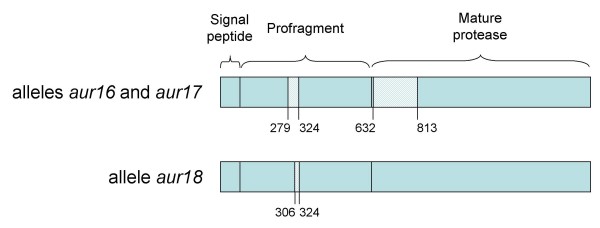
**Schematic representation of the recombinant segments in the *aur *gene**. The numbers under the gene are designated relative to the position of the first G in the GTG start codon of the *aur *gene and represent positions of recombination breakpoints.

Intragenic recombination events which result in a formation of linked runs of nucleotides within a sequence, whose ancestry is different from other nucleotides in the same sequence [19], are responsible for the mosaic structure of the sequence. In other words, a putative recombinant *aur *gene should consist of the DNA sequence stretches of both types of aureolysin. There was evidence of mosaicism within the *aur *alleles of type I for strains of ST22 (*aur16*), ST839 (*aur17*), and ST846 (*aur18*). Locations of breakpoints that represent end points of recombinant segments were found for alleles *aur16 *and *aur17 *pointing to recombination regions between nucleotides 279 and 324 and between 632 and 813, whereas for the allele *aur18 *data point to a region from nucleotides 306 to 324 (Figure 3).

After excision of the recombinant segments (228 nucleotides in total) from all *aur *sequences, the resulting 1302 bp fragments were compared. Pairwise differences in nucleotide sequences between representative pairs of shortened alleles ranged from 1 to 16 (1 to 40 for the whole gene) nucleotide sites within type I, and from 1 to 12 (1–13 for whole gene) nucleotide sites within type II. Therefore, after removal of the recombinant sequences, the polymorphism was substantially lower within type I, whereas within type II it was almost the same.

Very high congruence in topology was observed between phylogenetic trees based on the whole *aur *and shortened *aur *sequences (Figures 1B and 1C). This indicates that intragenic recombination had little effect on this gene. The only substantial differences were found for the branch lengths of recombinant *aur *alleles (*aur16*, *aur17*, and *aur18*).

### Biochemical comparison of two types of aureolysin

Purification to homogeneity by ammonium sulfate precipitation followed by ion exchange chromatography yielded 5 and 7 mg per liter culture for aureolysin of type I and type II, respectively. Enzyme titration with human α-2-macroglobulin showed 60% and 45% specific activity for types I and II, respectively, and this trend was observed in each preparation. N-terminal sequencing of the purified proteins revealed the AAATGT sequence for type I of the enzyme and an equimolar mixture of AAETGK and GKGVLG sequences for the type II (Figure 2). The latter sequences corresponded to putatively, properly processed and 6-amino acid truncated mature forms of aureolysin of type II, respectively.

A set of low molecular weight synthetic substrates differing at the C-terminus was used to compare the substrate specificity of both types of aureolysin. Fa-Gly-Ala-NH_2 _and Fa-Gly-Val-NH_2 _were barely hydrolyzed by the enzymes. By contrast, Fa-Gly-Phe-NH_2 _and Fa-Gly-Leu-NH_2 _were more susceptible to hydrolysis. Hence, basic kinetic studies were performed. Both enzymes exhibited almost identical K_M _and k_cat_/K_M _values for Fa-Gly-Phe-NH_2_. Similar data were acquired for Fa-Gly-Leu-NH_2_; however aureolysin of type I showed a slightly higher substrate affinity (4.4*10^-4 ^vs. 1.9*10^-3 ^M for type II). It is also worth noticing that Fa-Gly-Phe-NH_2 _is a better substrate than Fa-Gly-Leu-NH_2 _for both types of enzymes.

To confirm previous observations on substrate specificity, the synthetic substrate, galanin (29 amino acids), was used to test the substrate specificity of aureolysins. After 5 minutes incubation with the enzymes no differences in hydrolysis patterns were observed. Galanin, possessing the sequence: GWT|LNS|AG|Y|L|LGPHAIDNHRS|FHDKYG|LA-NH_2_, was digested (in places marked by |) at the N-terminal side of leucine, alanine, tyrosine and phenylalanine residues but not glycine, indicating that both types of aureolysin preferentially cleave peptide bonds before large hydrophobic amino acids. Additionally, selected proteins were incubated with both types of aureolysin and subjected to electrophoresis. In our hands no differences in the digestion patterns of β-casein, human low and high molecular weight kininogen, and human blood serpins, α-1-antitrypsin and α-1-antichymotrypsin, were observed. Moreover, aureolysin of type I alike type II had no proteolytic activity against elastin and type I collagen.

## Discussion

The value obtained for nucleotide diversity of *aur *(0.0554) revealed a high degree of gene polymorphism and was comparable to values obtained by Kuhn *et al*. [16] for core adhesion genes (mean = 0.077 ± 0.046) as well as accessory adhesion genes (mean = 0.088 ± 0.063). However, in contrast to findings by Kuhn *et al*. [16] that housekeeping and adhesion genes showed similar levels of purifying selection, our data revealed that the *aur *gene was subject to stronger purifying selection than the MLST genes. Furthermore, the least diversity and highest purifying selection of the gene fragment encoding the mature enzyme in comparison to the sequences coding for pre- and pro-fragment strongly suggest that proper enzymatic activity and specificity of aureolysin is very important in the processing of other staphylococcal proteins. Moreover, Beaufort and colleagues [10] recently reported that aureolysin can also be a highly specialized virulence factor. By precise proteolytic cleavage aureolysin activates the human fibrinolytic system, enabling *S. aureus *spread and invasion.

Although *S. aureus *is predominantly clonal, events of homologous exchange can occur and may play an important role in the evolution of *S. aureus *clones. Our results provide evidence for genetic exchange of the *aur *locus between unrelated strains of both supergroups and different clonal complexes. Kuhn and colleagues [16] previously showed high congruence between the gene trees constructed from concatenated sequences of MLST housekeeping, core adhesion (*clfA*, *clfB*, *fnbA*, *map*, *sdrC*, and *spa*), and accessory adhesion genes (*ebpS*, *fnbB*, *sdrD*, and *sdrE*), indicating that recombination had little effect on the population structure. However, when they investigated each gene separately, some evidence for homologous exchange was found. The best congruence between trees was observed with *clfA *and the least was observed with *clfB*. The *aur *locus is located close to the *clfB *locus (7 kb apart in all sequenced genomes). *S. aureus *is predominantly a clonal species, but some of its genomic regions show higher recombination rates and, based on the previous report of Kuhn and co-workers [16], we can infer that the *aur *locus is located in such a region, since it is located in the close vicinity of the *clfB *locus.

The restriction-modification (R-M) systems serve in phage defense as well as the stringent control in foreign DNA acquisition. Therefore, R-M systems are modulators of the frequency of genetic variation. Waldron and Lindsay [20] proposed that the Sau1 type I R-M system is the major mechanism for blocking gene transfer into *S. aureus *isolates from other species, as well as between isolates of different *S. aureus *lineages. Moreover, Sung and Lindsay [21] concluded that there are certain *S. aureus *lineages and strains possessing deficiencies in the dominant R-M what in turn leads to a hyperrecipient phenotype. They found naturally occurring hyperrecipient strains and the best example of this phenomenon was the bovine lineage ST151. Our analysis demonstrated that among the strains for which we found homologous transfer of the *aur *gene was also a strain of ST151. We can only hypothesize now that the same genetic mechanisms could play a role in exchange of the *aur *locus between the strains of other lineages.

Our analysis of the *aur *gene is esentially descriptive; however, the impact of the findings should be beneficial in designing future molecular tests. In our laboratory, based on knowledge about polymorphism of the *aur *gene, two rapid approaches are under construction for determination of the dominant *S. aureus *lineages and for species identification of the genus *Staphylococcus*. Currently, routine determination of *S. aureus *lineages is conducted by sequence-based typing methods (*spa *typing and MLST) or/and PFGE (Pulsed Field Gel Electrophoresis) which are time consuming and expensive. A new typing method will be based on restriction profile analysis of the *aur *gene by digestion with a restriction enzyme. However, there is confusion about the discriminatory power of a single marker (some unrelated lineages of supergroup II possess the same *aur *allele), therefore, another gene located in the genome at least 1 Mb from the *aur *gene (to eliminate a possibility that both genes took place in the same homologous exchange) will be chosen and investigated in detail. A new duplex PCR-RFLP (Restriction Fragment Length Polymorphism) method will be a substantial support for currently used typing methods in epidemiological studies.

Another molecular approach developing in our laboratory is single-tube multiplex-PCR for the simultaneous detection and discrimination of *Staphylococcus *species. For this purpose, genes encoding metalloproteinases from the thermolysin family are investigated. These genes are conserved among strains of given species and are unique compared to other species of the genus *Staphylococcus*. Primer pairs will be designed to yield single species-specific in size amplification products. Therefore, to design an efficient diagnostic test for *Stahylococcus *species identification, a very detailed knowledge about polymorphisms of the *aur *and *aur*-like genes is needed.

## Conclusion

In this paper we provide evidence that homologous recombination contributed to the evolution of the *aur *gene. Our study distinguishes between homologous exchanges of the entire *aur *gene (assortative recombination) between divergent lineages of *S. aureus *and recombination events within the *aur *gene. Despite the *aur *gene being highly polymorphic, purifying selection leads to limited variation of aureolysin at the amino acid level. Accordingly, purifying selection may provide only for small phenotypic differences and should not be a mechanism for isolating bacterial populations. Indeed, although we were able to detect minor differences in affinity of the proteases to low molecular weight synthetic substrates, no detectable dissimilarities in protein digestion pattern were observed. Mature forms of aureolysins whose biochemical properties were compared differed in 9 amino acid residues. Such a high degree of sequence similarity may mean overall structure identity, which in turn reflects no differences in protein digestion efficiency. However, keeping in mind the observed minor differences in the enzymatic properties against low molecular weight substrates, we cannot exclude a role for aureolysin in interactions with yet undefined proteins where two variants of this protease may act differently.

## Availability and requirements

NCBI:  

MLST: 

BURST:  

ClustalW: 

SNAP: 

## Competing interests

The authors declare that they have no competing interests.

## Authors' contributions

AJS partcipated in the study design, designed the primers, completed many of the amplifications, prepared part of the amplicons for sequencing, edited sequences, performed the alignments and phylogenetic analyses and wrote the manuscript. BW participated in the study design, biochemical analysis, wrote portions of the methods, results and discussion. KKS performed many of the amplifications, prepared the amplicons for sequencing. HG provided *S. aureus *strains and conducted critical assistance in drafting the manuscript. JMvD contributed to the revision of the manuscript. JK performed biochemical studies. PCA provided *S. aureus *strains and drafted the manuscript AD contributed in results analysis and was involved in drafting of the manuscript. WH provided *S. aureus *strains, participated in design and coordination of the project, and helped in drafting the manuscript. All authors read and approved the final manuscript.
